# The clinical relevance of microbiology specimens in orofacial abscesses of dental origin

**DOI:** 10.1308/003588412X13373405385539

**Published:** 2012-10

**Authors:** C Fowell, B Igbokwe, A MacBean

**Affiliations:** Shrewsbury and Telford Hospital NHS Trust,UK

**Keywords:** Orofacial, Dental infection, Abscess / drug therapy, Abscess / microbiology, Treatment outcome, Clinical relevance

## Abstract

**INTRODUCTION:**

It is common surgical practice to take a specimen for microbial culture and susceptibility (MC&S) when draining an orofacial abscess. The aim of this study was to determine if routine MC&S has any therapeutic value in the care of these patients.

**METHODS:**

A retrospective study was undertaken of records of patients admitted for surgical management of orofacial abscesses between January 2010 and December 2011. Records were reviewed for bacteriology specimen and result, admission details, antimicrobial treatment and outcome.

**RESULTS:**

A total of 79 patients were included in the study and specimens sent from 62 patients (78.4%). Samples were positive in 86.2% of cases, of which *Streptococcus viridans* was the most commonly isolated organism (54.7%). Interim reports were published on average after 3.25 days, with 89.9% of patients having been discharged within 2 post-operative days. According to clinical records, no patients in the cohort required further intervention or alteration of prescribed antimicrobial treatments following discharge.

**CONCLUSIONS:**

Almost 90% of patients were discharged before bacteriology results were available, without complication. This study suggests bacteriology culture has no therapeutic value in these patients. Omission of this practice in the case of uncomplicated orofacial abscesses could improve efficiency in the National Health Service without affecting patient care.

Along with extraction of the causative tooth, the incision and drainage of orofacial abscesses of dental cause is a common surgical procedure. This is often associated with the collection of a pus sample and its analysis for microbial culture and susceptibility (MC&S). Surgery is usually accompanied by the administration of broad-spectrum antibiotics and affords excellent prognosis in most cases. Many patients recover quickly and are discharged from hospital promptly.

The causative organisms of odontogenic infections have been described at length.^1,2^ However, there is little evidence on the therapeutic benefit of bacteriology sampling in these abscesses. The aim of this study was therefore to determine whether routine MC&S has any therapeutic value in patents treated for orofacial abscesses.

## Methods

A retrospective single centre cohort study was conducted of all patients admitted for incision and drainage of orofacial abscess under general anaesthesia between January 2010 and December 2011. Patients were identified from the trust surgical database. Data were collected from review of clinical records. Data included patient demographics, date of surgery and sample collection, culture results and antibiotic prescription. The dates of interim and final bacteriology results were compared with dates of admission, discharge and follow-up appointments.

## Results

Seventy-nine patients (41 male, 38 female) were admitted with orofacial abscesses and underwent incision and drainage during the study period. The mean patient age was 34.2 years (range: 5–74 years, median: 31 years). Forty-three patients (54.4%) were smokers. Eighteen (22.7%) were unemployed.

Thirty-two patients (40.5%) had drainage via a transcutaneous approach. The remainder (*n*=47, 59.5%) were drained intraorally. Microbiology samples were sent from 62 patients (78.4%). There was positive growth in 51 samples (82.3%). Of the 11 negative samples, 8 (72.7%) were from patients drained via a transcutaneous approach. The most commonly isolated organism from all cases was *Streptococcus viridans* (46.8%) (Table 1).

**Table 1:** Results of microbiological cultures

Isolated organism	Number of cases
*Streptococcus viridans*	29 (46.8%)
Coagulase negative staphylococci	17 (27.4%)
Anaerobes	12 (19.4%)
Beta-haemolytic streptococci	4 (6.5%)
*Staphylococcus aureus*	4 (6.5%)
*Haemophilus influenzae*	2 (3.2%)
*Moraxella catarrhalis*	2 (3.2%)
*Candida albicans*	2 (3.2%)
*Streptococcus milleri*	2 (3.2%)
*Enterococcus*	1 (1.6%)

Interim microbiology results were published in 86.2% of positive cases (*n*=44). On average, these results were made available 3.25 days (range: 0–8 days, median: 3 days) following collection.

Antibiotics were prescribed in for all patients (Table 2). They were prescribed intravenous antibiotics as inpatients and discharged to outpatient care with oral antibiotic therapy. Antibiotic regimens were not changed for any patient following publication of MC&S results. Two patients were prescribed antibiotics that would not have been effective in treating the reported bacterial culture results. Both patients were discharged prior to the microbiology result being available without a follow-up appointment. No complication was detected in these patients to our knowledge.

**Table 2:** Antibiotics prescribed during inpatient and outpatient care

Prescribed antibiotics (IV as inpatient, PO on discharge)	Number of cases
IV and PO amoxicillin and metronidazole	47 (59.4%)
IV and PO co-amoxiclav	10 (12.7%)
IV cefuroxime and metronidazole, PO amoxicillin and metronidazole	7 (8.9%)
IV clarithromycin and metronidazole, PO erythromycin and metronidazole	4 (5.1%)
IV cefuroxime and metronidazole, PO co-amoxiclav	3 (3.8%)
IV cefuroxime and metronidazole, PO erythromycin and metronidazole	3 (3.8%)
IV and PO co-amoxiclav and metronidazole	2 (2.5%)
IV benzylpenicillin and metronidazole, PO amoxicillin and metronidazole	1 (1.3%)
IV cefuroxime and metronidazole, PO metronidazole	1 (1.3%)
IV co-triximazole	1 (1.3%)

IV = intravenously; PO = *per os*

The mean length of post-operative stay was 2.0 days (range: 0–8 days, median: 1 day). Of the 44 patients for whom there was an interim report, 40 (90.9%) were discharged within 2 post-operative days.

Twenty-six patients (32.3%) were followed up in the outpatient department. No patients were readmitted for further intervention and none were prescribed further or alternative antimicrobial therapy.

## Discussion

Culture of microorganisms and testing for antimicrobial susceptibility is regarded as essential for the treatment of many infections. Along with incision and drainage and removal of the causative tooth, antibiotic therapy is the mainstay of treatment for orofacial abscesses of dental cause. However, in clinical practice, antimicrobial therapy is commenced empirically prior to results being available. In our study, the majority of patients were prescribed intravenous amoxicillin and metronidazole on admission. This was in keeping with local microbiology protocols. In the early stages of the study, intravenous co-amoxiclav was used although this was withdrawn from routine use by trust guidelines during the study period. Financial reasons were implicated.

The study shows no short-term complications following treatment with empirical antibiotic therapy and surgery. Almost 80% of patients treated had a bacteriology swab taken. The majority (90.9%) were discharged prior to any microbiology results being available. The microbial flora cultured from the specimens taken reflected documented and common organisms known to cause dental infections. The susceptibilities for these organisms were also predictable. This demonstrates the lack of therapeutic impact for these clinical investigations.

The culture and reporting of a microbiology specimen has been reported to cost £25–£30, not including the associated staff costs.^3^ For the 62 samples taken for this cohort, the approximate cost of analysis was £1,700.

The study is limited by its retrospective nature. It is difficult to ascertain how effectively microbiology results were reviewed. Hospital records and clinical case notes were reviewed but clinicians in the community may have undertaken further review of these results. Although no patients were readmitted or required further intervention following discharge, the possibility of a patient attending to his or her general practitioner or dental surgeon exists. The inclusion of data from primary care practitioners would have strengthened the results.

## Conclusions

The study demonstrates the lack of therapeutic value of routine bacteriology sampling in patients treated with surgery and empiric antibiotics for orofacial abscesses. We propose avoiding routine sampling in patients presenting with uncomplicated orofacial abscesses of dental origin. We did not encounter any patients with immunodeficiency, complicating co-morbidities or post-operative complications. We do not propose our conclusions are appropriate for such clinical scenarios.
